# Effect of Ultrasonic Waves and Other Technological Treatments on the Reduction of Acrylamide and Glycoalkaloids in Frozen Prefried Potatoes

**DOI:** 10.1155/ijfo/5685019

**Published:** 2026-04-24

**Authors:** Pedro G. Vargas-Finaflor, Elfer O. Obispo-Gavino, Sergio E. Contreras-Liza, Delsi M. Huaita Acha, Edwin A. Macavilca, Abraham G. Ygnacio Santa Cruz, Michelle Lozada-Urbano

**Affiliations:** ^1^ Department of Bromatology and Nutrition, Universidad Nacional José Faustino Sánchez Carrión, Huacho, Peru; ^2^ Academic Department of Agronomy, Universidad Nacional José Faustino Sánchez Carrión, Huacho, Peru; ^3^ General Directorate of Research, Universidad Inca Garcilaso de la Vega, Lima, Peru; ^4^ Academic Department of Food Industries Engineering, Universidad Nacional José Faustino Sánchez Carrión, Huacho, Peru; ^5^ Academic Department of Food Industries Engineering, Universidad Nacional Pedro Ruiz Gallo, Lambayeque, Peru; ^6^ Academic Programme in Nutrition and Dietetics, Universidad Privada Norbert Wiener, Lima, Peru

**Keywords:** acrylamide, glycoalkaloids, potato varieties, prefried frozen potatoes, response surface methodology, Taguchi design, ultrasound

## Abstract

The formation of toxic compounds in fried potato products is a major food safety concern due to their potential adverse effects on human health. This study aimed to evaluate the effects of processing and frying conditions on the contents of acrylamide (AC) and glycoalkaloids in three Peruvian potato varieties (Bicentenaria, CIP‐Poderosa Pollera, and Única). Samples were subjected to ultrasound pretreatment, variations in precooking and prefrying parameters, and three frying methods: deep‐frying (DF), oven‐frying (OF), and air‐frying (AF). A Taguchi design was applied to analyze color variation (Δ*E*), and a response surface methodology was used to optimize the processing parameters. The optimized treatment (OT1) corresponded to the Bicentenaria variety fried using the DF method (180°C, 6 min), with an ultrasonic amplitude of 28.93% of the maximum output of the 500 W system and prefrying at 77.10°C. This treatment achieved the highest desirability (0.88), low AC (19 µg/kg) and glycoalkaloid (6.97 mg/kg) contents, adequate hardness (7.50 N), and good color quality (Δ*E* = 14.83). Understanding optimal processing conditions and mitigation strategies is essential to produce safer potato products. The findings demonstrate that safe French fries can be obtained from local potato varieties such as Bicentenaria, representing a sustainable alternative to imported potatoes in the domestic industry.

## 1. Introduction

Potatoes constitute one of the most widely consumed foods in the Peruvian population and worldwide, with their use increasing in diverse product forms [1]. Among these, French fries stand out due to their high consumer acceptance, attributed not only to their crispness and pleasant flavor but also to their uniform color, characteristic aroma, and appropriate internal consistency. The quality of French fries is a key factor for consumers, who value the combination of physical and structural properties. Achieving this quality depends on selecting an appropriate potato variety, controlling cut thickness and uniformity, and applying a frying method that maintains a constant oil temperature, thereby preventing excessively greasy or overly browned products [2].

However, certain varieties with high levels of reducing sugars and low protein content may promote the formation of acrylamide (AC) when subjected to high temperatures (> 120°C) [3]. AC is a chemical compound naturally generated during intense thermal processes such as frying, baking, or roasting, and its presence is not associated with external contamination or product packaging [4]. Concerns regarding AC formation have led to the establishment of regulatory measures, particularly within the European Union, aimed at controlling cooking temperature and time [5]. The World Health Organization (WHO) warns that even low levels of exposure (< 500 µg/kg) may lead to long‐term adverse effects [6]. Reported risks include various types of cancer (breast, endometrial, ovarian, and kidney), genetic damage, and an increased likelihood of DNA mutations [7].

Recent studies have assessed the risk associated with AC consumption using novel approaches, identifying levels in French fries that may represent a significant risk to consumers [8]. Similarly, a study conducted in Italy reported a high mean AC concentration of 1116.6 ± 585 µg/kg in 50 French fry samples, with two brands exceeding recommended benchmark levels [9].

On the other hand, glycoalkaloids (GA) represent another group of compounds of health concern. These natural toxins are found in plants belonging to the Solanaceae family [10]. In potatoes, the main GA are α‐solanine and α‐chaconine, primarily located in the peel and flesh [10]. The WHO has established a maximum limit of 200 mg/kg of GA in potatoes intended for human consumption. Excessive intake may cause gastrointestinal symptoms, vomiting, diarrhea, fever, neurological alterations, and, in severe cases, potentially life‐threatening effects [11].

Recent research on tomato and potato plants has identified numerous biosynthetic genes involved in GA production [12]. This suggests the existence of genetic differences among potato varieties influencing the levels of these compounds, particularly the principal GA (α‐solanine and α‐chaconine). In potato tubers, the ratio between α‐chaconine and α‐solanine typically ranges from 1.2 to 2.6, indicating a generally higher presence of α‐chaconine, although this proportion depends on the variety used [13]. This difference is relevant due to the potential toxicity of GA, as their relative proportion may influence the health risk associated with potato consumption.

The application of emerging technologies such as ultrasound has gained increasing attention. Ultrasound has been employed as a pretreatment to enhance heat and mass transfer during food processing, particularly prior to frying. Several studies have demonstrated that its application, either alone or combined with treatments such as partial dehydration, can reduce frying time, improve color and texture, decrease oil absorption, and significantly reduce AC formation [14].

Ultrasound‐assisted frying (UAF) enables modification of pore networks and optimization of mass transfer pathways. This technology can reduce the processing time of French fries by 20%–50%, decrease energy consumption by 28%–39%, and reduce oil content by approximately 28.2%–31%. Furthermore, AC reductions of up to 90%–95% have been reported [15].

The consumption of French fries accounts for approximately 6%–46% of total dietary AC intake in many countries [6]. Therefore, the implementation of ultrasound and other pretreatments represents a promising strategy for producing safer and higher‐quality foods.

The objective of the present study was to evaluate three Peruvian potato varieties: Bicentenaria, CIP‐Poderosa Pollera, and Única. The Única variety is widely recognized for its suitability for frying; it is characterized by yellow flesh, a firm texture, and versatility, making it ideal for fried products [16]. The CIP‐Poderosa Pollera variety is a newly developed Peruvian cultivar specifically bred for frying, mainly cultivated in highland regions, and characterized by yellow flesh, a firm texture, and good frying performance [17]. In turn, the Bicentenaria variety is a recent development that has demonstrated excellent frying performance and a high content of functional components [18].

The production of frozen prefried potatoes was evaluated considering different processing factors, including ultrasound pretreatment at varying amplitude levels and varying precooking and frying temperatures and times, as well as three frying methods: deep frying, oven frying, and hot‐air frying. To analyze the effect of these factors on AC and glycoalkaloid formation and to optimize processing conditions, a Taguchi experimental design combined with response surface methodology (RSM) was applied, also considering color and texture parameters. This comprehensive approach enabled the identification of conditions that reduce undesirable compounds without compromising the final product quality.

## 2. Materials and Methods

### 2.1. Raw Materials

Three potato (*Solanum tuberosum* L.) varieties were used: Bicentenaria (clone UH‐24), Única (CIP392797.22), and CIP‐Poderosa Pollera (CIP395123.6), using 150 kg of each. The Bicentenaria and Única varieties were harvested from experimental fields in the district of Huaura (central coast of Peru), while CIP‐Poderosa Pollera was obtained from the central highlands. Harvesting was conducted during the first quarter of 2025 (January–March). The average environmental conditions during harvest were 22°C and 77% relative humidity. After harvesting, the tubers were stored for two weeks in a controlled environment (18°C–20°C, in the dark, with natural ventilation). Selection was performed manually to ensure physiological maturity, absence of physical defects, and uniformity in size. The potatoes were subsequently processed to obtain frozen prefried products. The prefried samples were stored at −18°C until final frying and subsequent physicochemical analyses.

### 2.2. Sample Preparation

The processing and frying of frozen prefried potatoes were conducted following the Peruvian Technical Standard NTP 011.124 [19]. Tubers were washed and manually peeled, with an average peeling depth of approximately 2–3 mm, performed uniformly across all samples. The tubers were then cut into sticks with a cross‐sectional dimension of 1 × 1 cm and a length ranging from 5 to 9 cm, depending on tuber size and potato variety. The cut samples were immersed in a citric acid solution (2 g/L) to prevent enzymatic browning.

Ultrasound treatment was performed using a QSONICA Q500 system (QSonica LLC, USA) at 20 kHz for 15 min. The amplitude was the variable parameter and was applied at 20%–40%, expressed as a percentage of the maximum output power of the equipment. A sample‐to‐water ratio of approximately 1:2 (200 g potato: 400 mL tap water) was used to ensure complete coverage of the sample and uniform propagation of ultrasonic waves throughout the medium.

**FIGURE 1:**
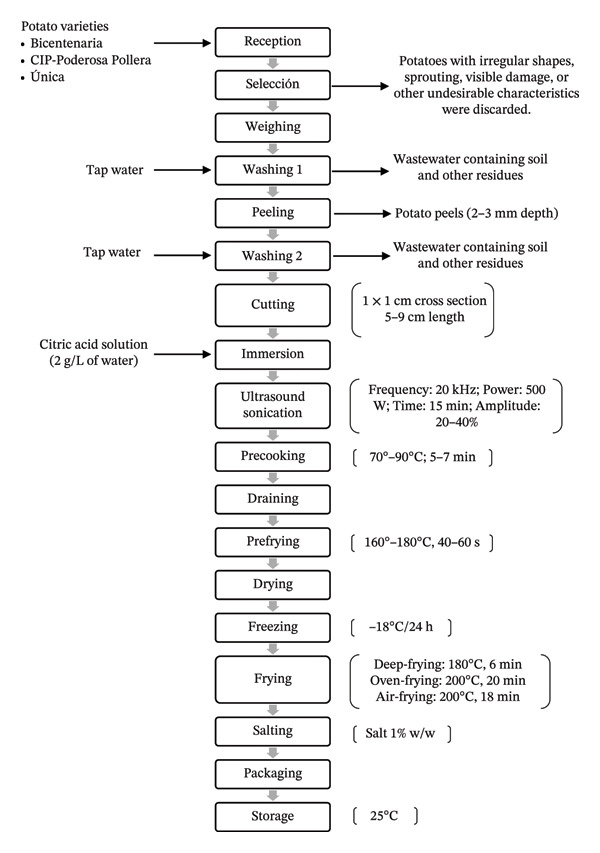
Process flowchart for the production of frozen prefried potatoes from three Peruvian varieties (Bicentenaria, CIP‐Poderosa Pollera, and Única), each processed independently under identical conditions.

Precooking (70°C–90°C, 5–7 min) and prefrying (160°C–180°C, 40–60 s) were then applied according to previously established procedures, with controlled temperature and time conditions [20, 21]. After air‐drying, the samples were packed in airtight bags and frozen at −18°C for 24 h. Subsequently, three final frying methods were evaluated: deep‐frying (DF) (180°C, 6 min), oven‐frying (OF) (200°C, 20 min, 4% oil), and air‐frying (AF) (200°C, 18 min, 4% oil), following standard protocols [22–24]. Finally, the fried potatoes were salted (1% w/w), packed, and stored at 25°C until further analysis (Figure 1).

All potato varieties were subjected to the same processing steps; however, different experimental treatments were applied according to the study design.

### 2.3. Proximate Analysis

The proximate composition of the raw materials (potato varieties Bicentenaria, CIP‐Poderosa Pollera, and Única) was determined at the Food Analysis Laboratory of Universidad Nacional José Faustino Sánchez Carrión (UNJFSC). Moisture, protein, fat, ash, and carbohydrate contents were measured according to standard AOAC methods. All results are expressed on a fresh weight (product) basis and reported as percentage (%).

#### 2.3.1. Moisture Analysis

Moisture content was determined using a RADWAG moisture analyzer (model MA 50/1.R) operating on the gravimetric principle by thermal drying, according to AOAC method 925.10 [25]. Approximately 5 g of homogenized sample, triturated with a mortar, was weighed directly on the instrument’s balance and subjected to automatic drying at a controlled temperature of 105°C until a constant weight was achieved.

#### 2.3.2. Protein Analysis

Crude protein content was determined by the micro‐Kjeldahl method according to AOAC 960.52 [26]. Approximately 1 g of dry sample per replicate was digested with sulfuric acid (H_2_SO_4_) in the presence of a catalytic mixture of potassium sulfate and cupric sulfate, using a DKL Kjeldahl digestion system (VELP Scientifica, Italy) at 420°C until a clear solution was obtained (typically 1–2 h). The digest was then distilled with concentrated sodium hydroxide (NaOH) in an automatic Kjeldahl distillation system (UDK 139, VELP Scientifica, Italy), and the released ammonia was collected in a 4% boric acid solution and titrated with 0.1 N hydrochloric acid (HCl) using a mixed indicator (methyl red). The total nitrogen content was converted to crude protein using a conversion factor of 6.25.

#### 2.3.3. Fat Analysis

The crude fat content was determined by the Soxhlet method according to AOAC 922.06 [27]. Approximately 3 g of dry, pulverized sample (triturated with a mortar) was weighed and placed in preconditioned extraction cartridges. The extraction was carried out using hexane as a solvent for 6 h in a conventional Soxhlet apparatus. At the end, the solvent was recovered by evaporation, and the collection flask was dried in an oven at 105°C for 1 h to remove possible residues. Subsequently, it was cooled in a desiccator and weighed. The fat content was calculated by weight difference.

#### 2.3.4. Ash Analysis

The ash content was determined according to AOAC 923.03 [28]. Approximately 3 g of dry, pulverized sample (triturated with a mortar after moisture removal) was weighed into preweighed porcelain capsules. The samples were initially placed in an oven at 105°C for 1 h to remove residual moisture and then incinerated in a Thermo Fisher Scientific furnace, model Thermolyne FB1410M, at 550°C for 6 h until white ash was obtained, indicating complete combustion of the organic matter. The capsules were then cooled in a desiccator and weighed. The ash content was calculated by weight difference.

#### 2.3.5. Carbohydrate Analysis

Total carbohydrate content was estimated by difference, according to AOAC 986.25 [29]. This determination was made by subtracting the previously analyzed percentages of moisture, protein, fat, and ash from 100%:

(1) Carbohydrate%=100−Protein%+fat%+ash%+moisture%.

### 2.4. pH Analysis

The pH was determined by the potentiometric method according to AOAC 981.12 [30]. For this purpose, 10 g of fresh sample was homogenized with 90 mL of distilled water (dilution 1:10, w/v) using a vortex mixer (VELP Scientifica, model VF 3). The mixture was measured with a digital potentiometer previously calibrated with buffer solutions of pH 4.0 and 7.0 at room temperature (∼25°C). Results are expressed on a fresh weight (product) basis and reported in pH units.

### 2.5. Titratable Acidity Analysis

Acidity was determined volumetrically and expressed on a fresh weight (product) basis and reported as a percentage citric acid equivalent [31]. For this purpose, 10 mL of the diluted extract, obtained by filtering the homogenized sample through filter paper, was titrated with 0.1 N NaOH using phenolphthalein as an indicator. The titratable acidity was calculated using the equation:

(2) Acidity%citric acid=VNaOH×NNaOH×PEVsample,

where

• *V*_NaOH_ = volume of NaOH spent in the titration(ml).
• *N*_NaOH_ = NaOH normality(0.1)
• PE = equivalent weight of citric acid(0.064g/meq).
• *V*_sample_ = titrated sample volume(mL)

### 2.6. Soluble Solids Analysis

The soluble solids content (°Brix) was determined by digital refractometry according to AOAC 932.12 [32]. For this purpose, 10 g of fresh sample was homogenized with 40 mL of distilled water (1:4, w/v) using a mortar, and the resulting mixture was filtered through Whatman filter paper. A drop of the filtrate was placed on the prism of an automatic digital refractometer (Lachoi, model LCH‐DR), previously calibrated with distilled water, and the reading was recorded at room temperature (∼25°C). The final soluble solids content was corrected for the dilution factor and expressed on a fresh weight (product) basis and reported as a percentage.

### 2.7. Analysis of Reducing Sugars

The determination of reducing sugars in the raw materials was performed using the colorimetric method with 3,5‐dinitrosalicylic acid (DNS) reagent [33]. For the calibration curve, a glucose stock solution was prepared, from which aliquots of 0.0, 0.1, 0.2, 0.4, 0.6, 0.8, 1.0, and 1.2 mL were transferred into labeled test tubes. To each tube, 1.0 mL of DNS reagent and an appropriate volume of distilled water were added to reach a final volume of 10 mL. The resulting glucose concentrations ranged from 0 to 130 ppm. A blank was prepared using 1.0 mL of DNS reagent and 9.0 mL of distilled water. All samples were heated in a water bath at 80°C for 6 min and then cooled under running tap water. Absorbance was measured at 540 nm using a PG Instruments UV–visible spectrophotometer, model T80+ (range: 190–1100 nm). The concentration of reducing sugars in the samples was determined by interpolation from the calibration curve. For the potato sample, 10 g of washed, peeled, and mashed tuber was homogenized and diluted with distilled water in a 100 mL beaker. From this solution, 1.0 mL was taken, mixed with 1.0 mL of DNS reagent and 8.0 mL of distilled water, and treated following the same procedure described for the standard solutions. Results are expressed on a fresh weight (product) basis and reported as a percentage (%).

### 2.8. Color Analysis

Color measurements of potato pulp and potato chips were performed using a CR‐400 colorimeter (Konica Minolta Inc., Japan), previously calibrated with a standard white plate. For potato pulp, color coordinates were recorded in the CIELab color space (L^∗^, a^∗^, b^∗^). For potato chips from local varieties, total color variation (Δ*E*) was determined relative to a commercial frozen prefried potato product (Bem Brasil brand) fried at 180°C for 6 min. Measurements were performed in triplicate for each treatment; Δ*E* was calculated using the following formula [34]:

(3) ΔE=L1∗−L2∗2+a1∗−a2∗2+b1∗−b2∗2,

where $L_{1}^{*},a_{1}^{*},b_{1}^{*}$ correspond to the values of each experimental treatment and $L_{2}^{*},a_{2}^{*},b_{2}^{*}$ to the values obtained from the commercial reference crisp (Bem Brasil brand), used as a comparative standard. L^∗^ represents the brightness (0 = black, 100 = white); a^∗^ indicates the scale from green (negative values) to red (positive values); and b^∗^ represents the scale from blue (negative values) to yellow (positive values).

### 2.9. Instrumental Hardness Analysis

The hardness of the potato chips was evaluated using a GÜSS Texture Analyzer (GÜSS Manufacturing Ltd., South Africa), previously calibrated according to the manufacturer’s specifications. The test was performed using a penetration/compression test, recording the hardness of each sample. Samples consisted of whole fried potato pieces, approximately 2 × 2 × 6 cm in size. A cylindrical probe (diameter 5 mm) was used, with a penetration speed of 8 mm/s and a penetration depth of 1 cm. The results were expressed in Newtons (N) as an indicator of the product’s resistance to the application of force.

### 2.10. AC Analysis

The analysis of AC in French fries was performed at the external laboratory ALAB Analytical Laboratory (Lima, Peru), accredited by the National Institute of Quality (INACAL) through Resolution No. 234‐2023‐INACAL/DA, and following the guidelines of the international standard ISO/IEC 17025:2017, according to the standardized method UNE‐EN 16618:2015 [35]. Approximately 2.0 g of the homogenized sample was mixed with 40 mL of water and an isotopically labeled internal standard (acrylamide‐d3, 1 µg/mL) to improve analytical accuracy and correct potential losses during sample preparation. After agitation and centrifugation, a 10 mL aliquot of the supernatant was recovered, purified by solid‐phase extraction, concentrated, reconstituted in 0.5 mL of water, and then filtered through a 0.45 µm membrane filter. AC was determined by liquid chromatography coupled to tandem mass spectrometry (LC–MS/MS), using a 10 µL injection volume and a 0.4 mL/min mobile phase flow. Identification was based on the retention time (≈3.5 min) in comparison with the internal standard and mass transitions in multiple reaction monitoring (MRM) mode. Results are expressed on a fresh weight (product) basis in µg/kg, and the method presented a limit of quantification of 5 µg/kg.

### 2.11. GA Analysis

The analysis of GA in French fries was expressed as α‐chaconine equivalents, since α‐chaconine is one of the principal GA present in potatoes [13], and was performed at the external laboratory ALAB Analytical Laboratory (Lima, Peru). Approximately 2.0 g of homogenized sample was extracted with 90% acetonitrile in water (v/v), vortexed for 1 min, agitated at 100 rpm for 15 min at room temperature, centrifuged at 3000 × g for 15 min, and the supernatant was filtered through a 0.2 µm PVDF membrane. α‐Chaconine was identified and quantified using a commercial standard, based on retention time (≈5–6 min) and mass transitions in MRM mode. Results are expressed on a fresh weight (product) basis in mg/kg α‐chaconine equivalents, and the method presented a limit of quantification of 0.005 mg/kg [36].

For potato raw material, GA were analyzed at the International Potato Center (CIP, Lima, Peru) and expressed as α‐chaconine equivalents. Approximately 10 g of washed and peeled tubers were homogenized into a uniform paste and hydrated with distilled water. GA were extracted using a methanol:chloroform solution, followed by phase separation with 2% acetic acid and petroleum ether, flocculation with ammonium hydroxide, and centrifugation to obtain a pellet containing α‐chaconine. The pellet was dissolved in 85% orthophosphoric acid, and absorbance was measured at 408 nm using a spectrophotometer. Quantification was performed by interpolation on a calibration curve prepared by mixing different volumes of the stock solution with orthophosphoric acid to obtain standard solutions with concentrations ranging from 10 to 100 ppm, and results are expressed on a fresh weight (product) basis in mg/kg of α‐chaconine equivalents [36].

### 2.12. Experimental Design and Statistical Analysis

The study was conducted in two phases. In the screening phase, a Taguchi L27 (3^5^) orthogonal design was applied. The Taguchi method accounts for uncontrollable factors that generate variability in experimental studies, minimizing losses from trial‐and‐error by using orthogonal matrices and regression models [11]. Five factors were considered: potato variety (*X*1), ultrasound power (*X*2), precooking (*X*3), prefrying (*X*4), and frying method (*X*5), with color variation (Δ*E*) as the response variable. An analysis of variance (ANOVA), followed by Tukey’s test (*p* < 0.05), was used to evaluate significant differences among treatments. All analyses were performed using Minitab Version 21 (Minitab LLC, USA). The factors and their corresponding levels are presented in Table 1.

**TABLE 1:** Levels of each factor according to the orthogonal arrangement L27 (3^5^).

Factor	Level		
	1	2	3
Potato variety	Bicentenaria	CIP‐Poderosa Pollera	Única
Ultrasound power (%)	20	30	40
Precooking (°C:min)	70:7	80:6	90:5
Prefrying (°C: s)	160:60	170:50	180:40
Frying method (°C:min)	Oven‐frying (200–20)	Deep‐frying (180–6)	Air‐frying (200–18)

In the optimization phase, a D‐optimal design with a quadratic model was developed in Design Expert 12 (Stat‐Ease Inc., USA). Optimal design usually refers to RSM, which evaluates the effects of multiple variables and their interactions, providing a mathematical model that predicts process behavior and facilitates optimization, determination of kinetic constants, and stability assessment in chemical and biochemical systems [37].

In this second phase, independent variables with the lowest *p* values identified during the initial screening were retained for inclusion in the optimization, while the factor with the highest *p* value, deemed statistically nonsignificant in the first phase, was kept constant.

The response variables considered were color variation (Δ*E*), hardness (*N*), AC (µg/kg), and GA (α‐chaconine, mg/kg). The *D*‐optimal design generated combinations of four independent variables (*X*1–*X*4), coded as continuous (−1 to +1) and categorical (Levels 1 and 2), to evaluate their effect on the response variables (*Y*1 = Δ*E*, *Y*2 = hardness, *Y*3 = AC, *Y*4 = GA). The design and factor levels are summarized in Table 2.

**TABLE 2:** *D*‐optimal design for the optimization of frozen prefried potatoes.

T	Independent variable			
	*X*1 (ultrasound power)	*X*2 (precooking)	*X*3 (prefrying)	*X*4 (frying method)
1	0.35	−1.00	Level 1	Level 1
2	−0.70	−0.70	Level 1	Level 1
3	−1.00	0.35	Level 1	Level 1
4	1.00	1.00	Level 1	Level 1
5	−1.00	−1.00	Level 2	Level 1
6	1.00	−1.00	Level 2	Level 1
7	0.23	−0.95	Level 2	Level 1
8	−1.00	1.00	Level 2	Level 1
9	−1.00	−1.00	Level 1	Level 2
10	1.00	−0.33	Level 1	Level 2
11	−0.35	1.00	Level 1	Level 2
12	0.34	−1.00	Level 2	Level 2
13	−1.00	0.35	Level 2	Level 2
14	−1.00	0.35	Level 2	Level 2
15	1.00	1.00	Level 2	Level 2

*Note:* T = treatment; *X*1–*X*4 = independent variables considered in the *D*‐optimal design.

During treatment optimization, quantities were assigned, and targets were set to maximize, minimize, or maintain the studied ranges of each response variable. All treatments were evaluated according to these criteria, and the resulting desirability values were calculated. Subsequently, the three most promising treatments, presenting the highest desirability values, were selected. The validity of the models was verified using ANOVA (*p* < 0.05) and the coefficients of determination (*R* ^2^ and adjusted *R* ^2^).

## 3. Results and Discussion

### 3.1. Color Analysis

Color analysis was performed on the pulp of the potato varieties evaluated. The Única variety showed greater lightness (*L*) compared to the others. On the other hand, the Bicentenaria variety showed more negative values on the a^∗^ axis and more positive values on the b^∗^ axis, indicating a greater tendency toward greenish and yellowish tones, respectively.

Bicentenaria potato: color (L^∗^a^∗^b^∗^) (64.12 ± 0.41; −5.87 ± 0.82; 37.48 ± 1.24); CIP‐Poderosa Pollera potato (61.13 ± 0.21; −3.56 ± 0.12; 34.37 ± 0.57); Única potato (65.52 ± 1.32; −3.29 ± 0.14; 28.04 ± 0.61).

These color differences between varieties may be influenced by the presence and concentration of phenolic compounds and antioxidants. These secondary metabolites are found in both the skin and the flesh, and their content varies according to genotype, flesh color, stage of development, and storage conditions. Potatoes with white pulp tend to have lower levels of carotenoids than those with yellow or orange pulp, which would explain the greater lightness observed in the Única variety compared to the others [38].

In addition, the reducing sugars present in the tuber also influence color development, especially during frying processes, due to non‐enzymatic browning reactions. Although this analysis was performed on fresh pulp, the initial differences in reducing sugar content between varieties (Table 3) could contribute to the variations in hue detected [39].

**TABLE 3:** Physicochemical and chemical composition of raw potato varieties (mean ± SD).

Parameter	Units	Bicentenaria	CIP‐Poderosa Pollera	Única
Soluble solids	°Brix	5.17 ± 0.06^a^	5.23 ± 0.06^a^	4.80 ± 0.26^b^
pH	—	6.11 ± 0.01^b^	6.08 ± 0.01^c^	6.26 ± 0.01^a^
Titratable acidity	% citric acid	0.15 ± 0.00^a^	0.15 ± 0.00^a^	0.14 ± 0.00^b^
Reducing sugars	%	0.02 ± 0.00^b^	0.06 ± 0.00^a^	0.02 ± 0.00^b^
Glycoalkaloids (α‐chaconine)	mg/kg	14.60 ± 3.30^b^	85.30 ± 2.90^a^	1.80 ± 0.30^c^

*Note:* Different superscript letters in the same row indicate significant differences (*p* < 0.05, Tukey’s test).

### 3.2. Analysis of the Physicochemical and Chemical Characteristics of Potato Varieties

A difference was observed in the reducing sugar content among the varieties evaluated, with CIP‐Poderosa Pollera having the highest value (0.06%), while Bicentenaria and Única showed lower levels (0.02%) (Table 3). This variability can be attributed to agroclimatic conditions, as tubers grown in high Andean areas tend to accumulate higher concentrations of reducing sugars, which increases the risk of AC formation during thermal processes. From an industrial perspective, cultivars with low reducing sugar content are preferred by the potato chip and crisp industry, as they favor a lighter color and reduce the formation of unwanted compounds derived from the Maillard reaction [40, 41].

In terms of α‐chaconine content, all varieties had values well below the recommended maximum limit of 200 mg/kg for total GA in fresh potatoes (Moyo et al.) [42]. CIP‐Poderosa Pollera showed the highest concentration (85.30 mg/kg), while Bicentenaria and Única recorded lower levels (14.60 and 1.80 mg/kg, respectively). These differences may be related to genetic and environmental factors, especially exposure to UV radiation and abiotic stress characteristic of high Andean areas, which induce the biosynthesis of secondary metabolites such as α‐chaconine [38]. Although the concentrations found do not represent a toxicological risk, values above 100 mg/kg could affect the marketability of the product [39].

On the other hand, Table 4 shows the results of the proximate analysis of the potato varieties evaluated. It was observed that moisture was high in all samples, carbohydrates were the major component, and proteins, fats, and ash were found in lower proportions.

**TABLE 4:** Proximate composition of raw materials.

Parameters	Bicentenaria	CIP‐Poderosa Pollera	Única
Moisture (%)	76.27 ± 0.85^a^	73.78 ± 0.92^b^	75.14 ± 0.78^ab^
Ash (%)	0.97 ± 0.05^a^	0.76 ± 0.04^b^	0.44 ± 0.03^c^
Fat (%)	0.30 ± 0.02^b^	0.34 ± 0.03^ab^	0.39 ± 0.04^a^
Protein (%)	2.10 ± 0.08^b^	2.29 ± 0.10^a^	2.19 ± 0.09^ab^
Carbohydrates (%)	20.36 ± 0.82^b^	22.83 ± 0.88^a^	21.84 ± 0.79^ab^

*Note:* Different superscript letters in the same row indicate significant differences (*p* < 0.05, Tukey’s test).

The moisture content ranged between 73.78% and 76.27%. These results coincide with those reported by Moyo et al. [42], who indicate that the moisture content of potato tubers usually ranges between 63% and 87%, depending on the variety and growing conditions. Likewise, it was observed that moisture values are related to dry matter content, which is consistent with the findings of Alva et al. [43], who indicate that the dry matter range for potatoes intended for frying is 22%–29%. In this study, the calculated dry matter content ranged from 23.73% to 26.22%, falling within that range.

With regard to protein content, the values ranged from 2.10% to 2.29%, coinciding with the findings of Moyo et al. [42], who reported levels close to 2% in potatoes from Uganda and slightly higher in samples from Kenya. The low fat content (0.30%–0.39%) confirms that potato tubers are naturally low in lipids, as noted by the same authors, who report values at the lower end of the range for other varieties.

Ash content ranged from 0.44% to 0.97%, values within the interval reported by Moyo et al. [42] (0.62%–1.36%). Carbohydrate content varied between 20.36% and 22.83%, slightly exceeding the range also reported by the same authors [42] (12.9%–19.6%). These differences may be attributed to varietal characteristics, environmental conditions, and variations in dry matter accumulation, as carbohydrates represent the main component of potato tubers and are strongly influenced by genotype and cultivation practices.

### 3.3. Stage I: Screening of Factors Affecting Color Variation

#### 3.3.1. Taguchi Design

The results obtained from the 27 experimental treatments revealed that the CIP‐Poderosa Pollera variety exhibited the highest Δ*E* values, indicating greater color variability compared to the commercial Bem Brasil potato, particularly with certain frying methods such as DF and AF. In contrast, the Única and Bicentenaria varieties showed lower Δ*E* values in several treatments, suggesting less color variability during the frying process (Table 5).

**TABLE 5:** Taguchi L27 orthogonal design and corresponding Δ*E* values.

T	*X*1	*X*2	*X*3	*X*4	*X*5	*Y*1	
	Potato variety	Ultrasound power (%)	Precooking (°C:min)	Prefried (°C:s)	Frying method (°C:min)	Δ*E*	SD
1	Bicentenaria	20	70:7	160:60	Oven‐frying	21.38	±2.70
2	Bicentenaria	20	70:7	160:60	Deep‐frying	16.43	±10.90
3	Bicentenaria	20	70:7	160:60	Air‐frying	20.64	±2.58
4	Bicentenaria	30	80:6	170:50	Oven‐frying	21.22	±1.17
5	Bicentenaria	30	80:6	170:50	Deep‐frying	21.08	±2.25
6	Bicentenaria	30	80:6	170:50	Air‐frying	26.25	±2.49
7	Bicentenaria	40	90:5	180:40	Oven‐frying	18.00	±1.86
8	Bicentenaria	40	90:5	180:40	Deep‐frying	20.10	±0.76
9	Bicentenaria	40	90:5	180:40	Air‐frying	21.33	±2.62
10	CIP‐Poderosa Pollera	20	80:6	180:40	Oven‐frying	16.92	±1.25
11	CIP‐Poderosa Pollera	20	80:6	180:40	Deep‐frying	43.35	±1.11
12	CIP‐Poderosa Pollera	20	80:6	180:40	Air‐frying	31.35	±3.03
13	CIP‐Poderosa Pollera	30	90:5	160:60	Oven‐frying	26.47	±4.80
14	CIP‐Poderosa Pollera	30	90:5	160:60	Deep‐frying	56.66	±1.79
15	CIP‐Poderosa Pollera	30	90:5	160:60	Air‐frying	59.32	±2.60
16	CIP‐Poderosa Pollera	40	70:7	170:50	Oven‐frying	22.50	±4.11
17	CIP‐Poderosa Pollera	40	70:7	170:50	Deep‐frying	20.20	±1.91
18	CIP‐Poderosa Pollera	40	70:7	170:50	Air‐frying	29.52	±6.14
19	Única	20	90:5	170:50	Oven‐frying	9.38	±1.81
20	Única	20	90:5	170:50	Deep‐frying	12.74	±1.87
21	Única	20	90:5	170:50	Air‐frying	20.32	±5.18
22	Única	30	70:7	180:40	Oven‐frying	9.85	±6.18
23	Única	30	70:7	180:40	Deep‐frying	18.80	±0.42
24	Única	30	70:7	180:40	Air‐frying	20.33	±2.62
25	Única	40	80:6	160:60	Oven‐frying	5.08	±2.35
26	Única	40	80:6	160:60	Deep‐frying	12.25	±2.28
27	Única	40	80:6	160:60	Air‐frying	15.84	±1.28

*Note:* T = treatment; Δ*E* = color variation.

Abbreviation: SD, standard deviation.

**FIGURE 2:**
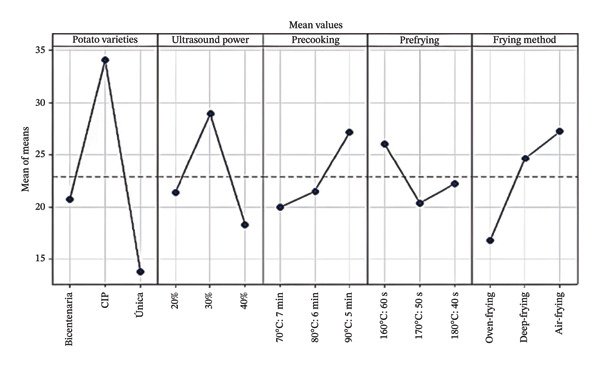
Effects of factors on color variation (Δ*E*) in frozen prefried potatoes. Points represent mean Δ*E* values for each factor level.

ANOVA indicated that potato variety (*p* < 0.001), ultrasound power (*p* = 0.014), and frying method (*p* = 0.014) had significant effects on color variation (Table 6). The prefrying factor showed a higher *p* value (0.236), reflecting lower statistical significance. The levels with the highest mean Δ*E* values—specifically the CIP‐Poderosa Pollera variety and the AF method—were excluded from the subsequent optimization phase due to their high color variation. The results are presented in Figure 2.

**TABLE 6:** Analysis of variance (ANOVA) of the main factors affecting color variation.

Source	GL	SC sec.	SC adjust.	MC adjust.	*F*‐value	*p* value
Potato variety	2	1896.30	1896.30	948.14	20.13	< 0.01^∗∗^
Ultrasound	2	532.30	532.30	266.13	5.65	0.014^∗^
Precooking	2	258.20	258.20	129.09	2.74	0.095 ns
Prefrying	2	149.20	149.20	74.62	1.58	0.236 ns
Method	2	533.50	533.50	266.77	5.66	0.014^∗^
Residual error	16	753.50	753.50	47.10		
Total	26	4123.00				

*Note:* GL: degrees of freedom; SC Sec: sum of squares; SC adjust.: adjusted sum of squares; MC adjust.: adjusted mean square. Significance levels: ^∗^ *p* < 0.05; ^∗∗^ *p* < 0.01; ns, not significant (*p* > 0.05).

These results contrast with those reported by Verma et al. [44], who highlighted air frying as an effective method for improving product quality by significantly reducing AC formation. However, in this study, color variation (Δ*E*) was evaluated, and the results suggest that, under our experimental conditions, air frying did not promote color uniformity. This behavior may be influenced by factors inherent to the raw material, such as reducing sugar content or initial moisture, as well as by prior ultrasound and precooking treatments, which could have intensified non‐enzymatic browning reactions during frying.

### 3.4. Stage II: Optimization Using RSM

The experimental results obtained from the combinations generated using the D‐optimal design are presented. The factors and levels included in the optimization phase were selected based on the screening analysis, in which the prefrying factor was excluded due to its high *p* value (0.236), indicating low statistical significance. The four factors considered in the treatments were *X*1 = ultrasound power (20%–30%), *X*2 = precooking temperature (70°C–80°C), *X*3 = potato variety (Bicentenaria and Única), and *X*4 = frying method (deep frying and oven frying). Table 2 illustrates the experimental layout used in the optimization phase, showing the methodology for selecting factor combinations and their levels, facilitating the understanding of the treatment combinations. Table 7 summarizes the experimental results obtained for the response variables: Δ*E*, instrumental hardness, AC content, and glycoalkaloid concentration (α‐chaconine).

**TABLE 7:** Experimental responses obtained from the *D*‐optimal design.

Treatment	Independent variable				Dependent variable			
	Ultrasound power (%)	Precooking (°C)	Potato variety	Frying method	ΔE	Hardness (*N*)	Acrylamide (µg/kg)	Glycoalkaloids (α‐chaconine) (mg/kg)
1	26.75	70.00	Bicentenaria	Oven‐frying	14.16	9.70	19.00	42.95
2	21.50	71.50	Bicentenaria	Oven‐frying	14.06	9.13	20.00	33.38
3	20.00	76.75	Bicentenaria	Oven‐frying	15.25	7.07	15.00	6.20
4	30.00	80.00	Bicentenaria	Oven‐frying	15.06	10.75	21.00	6.56
5	20.00	70.00	Única	Oven‐frying	16.57	6.63	37.00	0.55
6	30.00	70.00	Única	Oven‐frying	17.17	9.25	41.00	0.43
7	26.15	70.23	Única	Oven‐frying	15.14	8.50	30.00	0.46
8	20.00	80.00	Única	Oven‐frying	17.26	8.16	45.00	0.51
9	20.00	70.00	Bicentenaria	Deep‐frying	17.06	7.42	12.00	19.24
10	30.00	73.35	Bicentenaria	Deep‐frying	15.38	9.27	21.00	6.18
11	23.25	80.00	Bicentenaria	Deep‐frying	15.90	4.72	24.00	19.81
12	26.70	70.00	Única	Deep‐frying	17.25	10.64	37.00	1.60
13	20.00	76.75	Única	Deep‐frying	15.60	10.41	36.00	2.52
14	20.00	76.75	Única	Deep‐frying	15.59	11.12	37.00	2.42
15	30.00	80.00	Única	Deep‐frying	16.15	10.59	30.00	1.96

*Note:* Δ*E*: color variation.

#### 3.4.1. Color Variation (Δ*E*)

The ANOVA showed that the quadratic model for Δ*E* was statistically significant (*p* < 0.05), with a coefficient of determination (*R* ^2^) of 0.997 and an adjusted *R* ^2^ of 0.979, confirming that the model adequately described the data (Table 8). Among the factors evaluated, potato variety (*p* < 0.05) and frying method (*p* < 0.05) had significant effects on color variation, as did their interaction (*p* < 0.05).

**TABLE 8:** Statistical parameters for the model adequacy of color variation (Δ*E*).

Parameter	Value
Standard deviation	0.15
Mean	15.84
Coefficient of variation (%)	0.96
*R* ^2^	0.997
Adjusted *R* ^2^	0.979
Adequate precision	22.72

Figure 3 shows the contour plot (a) and the three‐dimensional response surface (b), illustrating Δ*E* variation as a function of ultrasound power (*X*‐axis) and precooking temperature (*Y*‐axis) in deep‐fried Bicentenaria potatoes, under optimal categorical conditions determined using the *D*‐optimal design. In both cases, the color scale represents Δ*E* values, with blue indicating smaller color differences and red indicating larger deviations from the optimal product. The lowest Δ*E* values (∼14.06) were observed in the central region of the graph, corresponding to ultrasound powers between 24% and 30% and precooking temperatures of 74°C–78°C, while combinations such as 20% ultrasound and 70°C precooking resulted in higher Δ*E* values (∼17.26). These results suggest that intermediate ultrasound powers and moderate precooking temperatures favor color retention in the final product.

FIGURE 3Contour plot (a) and response surface (b) for Δ*E* of French fries as a function of ultrasound power and precooking temperature in deep‐fried Bicentenaria potatoes. Blue regions indicate lower Δ*E* (closer to commercial reference).

**Figure:**
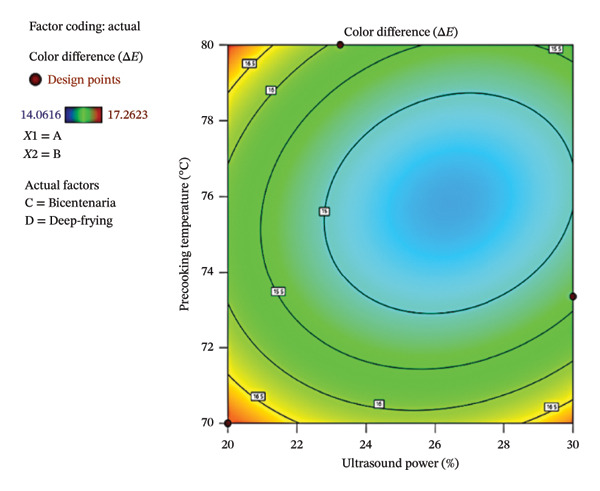
(a)

**Figure:**
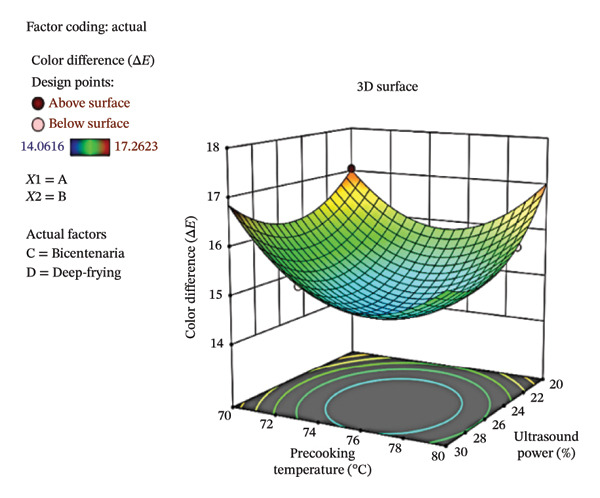
(b)

This behavior is partially consistent with the findings of Antunes‐Rolin et al. [45], who evaluated the effect of ultrasound on potatoes during prefrying hydration. They reported that higher ultrasonic power (100%) combined with a lower hydration temperature (42°C) resulted in higher L^∗^ values (lighter samples) and lower a^∗^ values (less reddish hue). These changes in color parameters were associated with lower AC formation, attributed to greater moisture retention and reduced concentration of reducing sugars on the tuber surface prior to frying. Although the specific optimal conditions differ, both studies indicate that ultrasound‐assisted treatments can improve color and reduce undesired chemical reactions during frying.

#### 3.4.2. Hardness

The ANOVA indicated that the overall model for the hardness variable was not statistically significant (*p* > 0.05). However, the interaction between potato variety and frying method showed a significant effect (*p* < 0.05). The lack‐of‐fit analysis was not significant (*p* > 0.05), supporting the validity of the model, while the regression parameters (*R* ^2^ = 0.968 and adjusted *R* ^2^ = 0.774) indicate an adequate fit to the experimental data (Table 9).

**TABLE 9:** Statistical parameters for the model adequacy of hardness.

Parameter	Value
Standard deviation	0.87
Mean	8.89
Coefficient of variation (%)	9.72
*R* ^2^	0.968
Adjusted *R* ^2^	0.774
Adequate precision	7.55

Figure 4 presents the contour plot (a) and three‐dimensional response surface (b), illustrating the influence of ultrasound power and precooking temperature on the hardness of deep‐fried Bicentenaria potatoes. The highest hardness values were observed at ultrasound powers of 28%–30% and precooking temperatures of 70°C–72°C, corresponding to greater product firmness, whereas low ultrasound power (< 26%) combined with high precooking temperatures (> 78°C) resulted in lower hardness values. Overall, hardness ranged from 4.72 to 11.12 N, partially within and partially outside the optimal range of 7–10 N reported by Ahmed et al. [46]. Although DF and OF produced values across this range, the global model did not reveal significant differences (*p* > 0.05), suggesting that other factors, such as initial moisture content, crust formation, or dry matter, may have a stronger influence on hardness than the parameters evaluated in this study.

FIGURE 4Contour plot (a) and response surface (b) for hardness (*N*) as a function of ultrasound power and precooking temperature in deep‐fried Bicentenaria potatoes.

**Figure:**
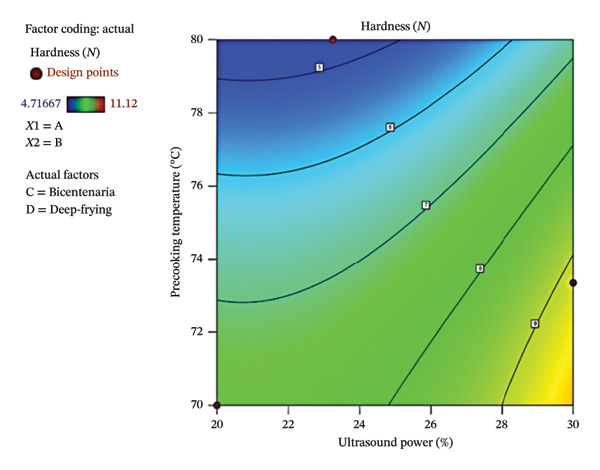
(a)

**Figure:**
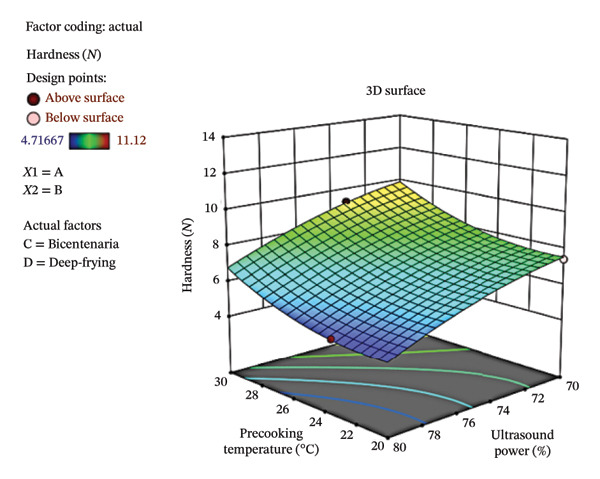
(b)

#### 3.4.3. AC

The ANOVA showed that the overall model for the AC variable was not statistically significant (*p* > 0.05). However, it was identified that the potato variety had a significant effect (*p* < 0.05), which demonstrates its influence on the formation of AC. Other factors such as ultrasound power, precooking, and frying method did not show significant effects (*p* > 0.05). Despite this, the regression parameters (*R* ^2^ = 0.967 and adjusted *R* ^2^ = 0.768) show an adequate fit to the experimental data (Table 10).

**TABLE 10:** Statistical parameters for the model adequacy of acrylamide.

Parameter	Value
Standard deviation	4.91
Mean	28.33
Coefficient of variation (%)	17.32
*R* ^2^	0.967
Adjusted *R* ^2^	0.768
Adequate precision	6.85

Figure 5 shows the contour plot (a) and three‐dimensional response surface (b), where the influence of ultrasound power and precooking temperature on the AC concentration in deep‐fried Bicentenaria potatoes can be observed. Low AC values were found in the range of 20%–24% ultrasound power and 70°C–74°C precooking temperature, conditions that were favorable for minimizing the formation of this toxin. In contrast, high AC levels were observed at ultrasound powers above 28% and temperatures close to 80°C, suggesting that these combinations intensify AC formation.

FIGURE 5Contour plot (a) and response surface (b) for acrylamide (µg/kg) as a function of ultrasound power and precooking temperature in deep‐fried Bicentenaria potatoes.

**Figure:**
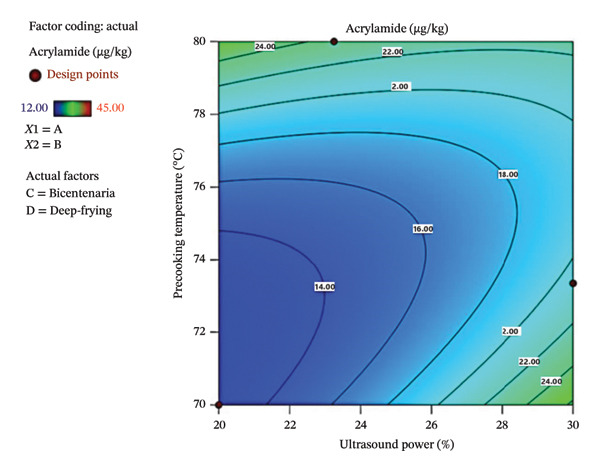
(a)

**Figure:**
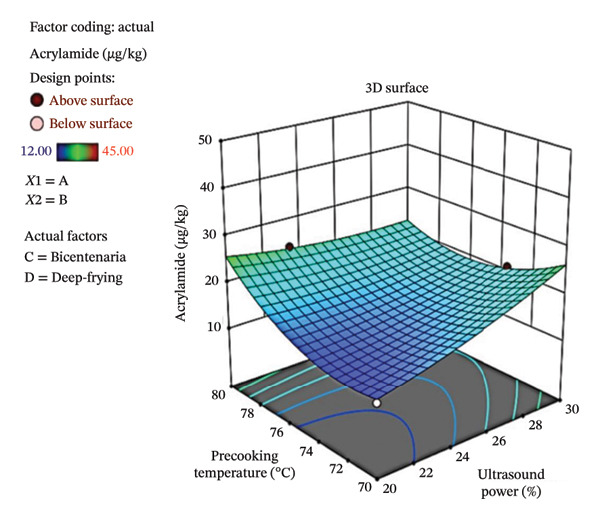
(b)

The AC levels obtained in the French fries were considerably low, with values between 12 and 45 µg/kg, well below the reference value established by the European Commission regulation [44] of 500 µg/kg for French fries. This low AC formation could be attributed to the low reducing sugar content in both varieties evaluated (Bicentenaria and Única), with an average of 0.02%, which limits the substrate available for the Maillard reaction with asparagine during heat treatment. The use of ultrasonic precooking pretreatments, applied at different powers and temperatures, probably favored the reduction of AC, as reported by Antunes‐Rohling et al. [45], who observed reductions of up to 90% in AC after applying high‐intensity ultrasound under controlled conditions of temperature (42°C) and time (30 min). This effect is associated with ultrasonic cavitation, which promotes the leaching of reducing sugars, thus reducing the availability of precursors for the Maillard reaction. Regarding the effect of the frying method, both DF (180°C/6 min) and OF (200°C/20 min) generated similar levels of AC, although a slight increase was observed in the OF, as shown in Table 7. This finding contrasts with that reported by Verma et al. [44], who found that hot air methods reduced AC formation compared to DF in oil, and differs partially from that reported by Navruz & Mortaş [23], who observed lower values in baked potatoes (7 µg/kg) compared to fried potatoes (9 µg/kg), attributing this reduction to moisture control.

#### 3.4.4. GA

The ANOVA showed that the general model for GA was statistically significant (*p* < 0.05). Among the factors, only the potato variety had a significant effect (*p* < 0.05), while ultrasound power, precooking, and frying method showed no statistical influence. However, the model showed an excellent fit, with a high coefficient of determination (*R* ^2^ = 0.994) and an adjusted *R* ^2^ of 0.955 (Table 11), demonstrating an adequate fit to the experimental data.

**TABLE 11:** Statistical parameters for the model adequacy of glycoalkaloids.

Parameter	Value
Standard deviation	2.82
Mean	9.65
Coefficient of variation (%)	29.27
*R* ^2^	0.994
Adjusted *R* ^2^	0.955
Adequate precision	17.01

Figure 6 shows the contour plot (a) and three‐dimensional response surface (b) showing the variation in GA concentration as a function of ultrasound power and precooking temperature in deep‐fried Bicentenaria potatoes. High GA values were found in the range of 22%–28% ultrasound power and at low precooking temperatures (70°C–72°C). In contrast, low GA contents were recorded when ultrasound powers were high (28%–30%) and precooking temperatures were moderate (74°C–80°C), where the surface acquires blue tones.

FIGURE 6Contour plot (a) and response surface (b) for glycoalkaloids (α‐chaconine) as a function of ultrasound power and precooking temperature in deep‐fried Bicentenaria potatoes.

**Figure:**
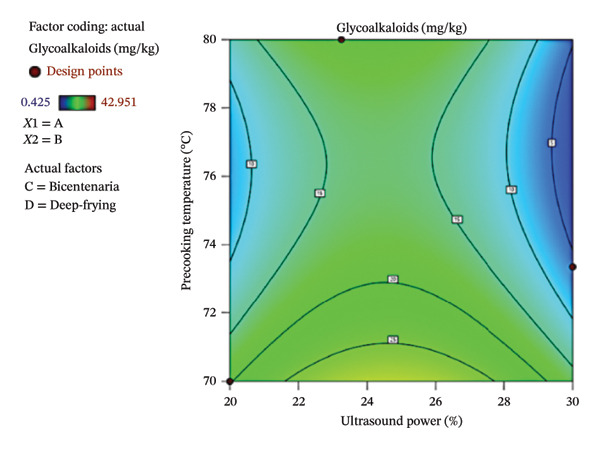
(a)

**Figure:**
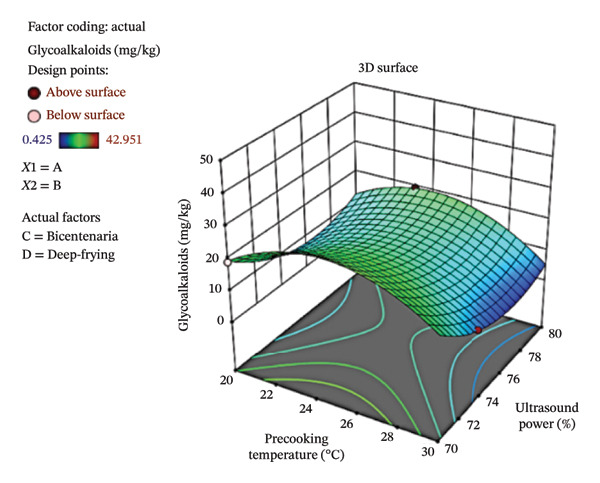
(b)

When compared with the initial contents of 14.6 mg/kg in Bicentenaria and 1.8 mg/kg in Única, it can be seen that the latter showed very low levels of GA in potato chips (0.43–2.52 mg/kg), while Bicentenaria had a higher content (6.18–42.95 mg/kg), which shows varietal differences in thermostability and glycoalkaloid elimination. These results coincide with those reported by Takagi et al. [47], who described that α‐chaconine maintains high stability in moderate cooking processes and only partially degrades at temperatures above 210°C. Complementarily, Koyuncu & Duran [11] demonstrated that more intense frying parameters with short times (165 s) and high temperatures (190°C) significantly reduce the glycoalkaloid content, with frying time being the factor with the greatest impact (76.5% decrease). It should be noted that, although in some treatments the concentrations were higher than the initial ones, in all cases they remained well below the indicative toxicity level established by the European Commission [48], set at 100 mg/kg, confirming that the processes evaluated allow GA to be kept within safe ranges for consumers.

#### 3.4.5. Desirability Analysis

**FIGURE 7:**
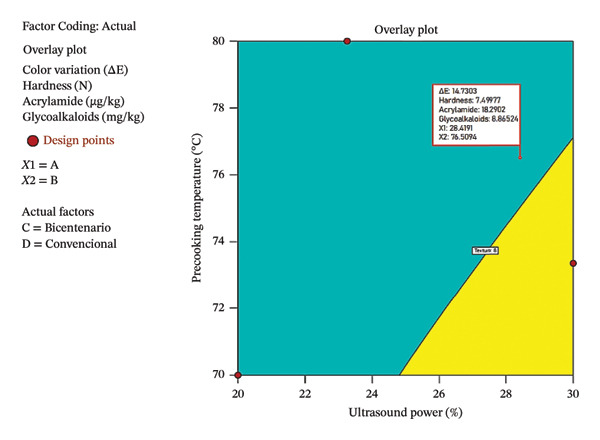
Overlay plot showing the region of simultaneous optimization for Δ*E*, hardness, acrylamide, and α‐chaconine in deep‐fried Bicentenaria potatoes.

Figure 7 shows the overlay graph generated by Design Expert software, which represents the areas of simultaneous compliance of the response variables. This graph was constructed based on ultrasound power (*A*) and precooking temperature (*B*), keeping the categorical variables corresponding to the Bicentenaria variety and the DF method constant.

The light blue shaded region represents the area where the four response variables meet the established desirable ranges, while the yellow area indicates experimental conditions outside the optimal range. The point marked on the graph corresponds to the optimal treatment (OT1) proposed by the model, and under these conditions, the values of Δ*E*, hardness, AC, and GA (α‐chaconine) are predicted.

Table 12 presents the desirability analysis to determine the optimal treatments considering different combinations of ultrasound power, precooking temperature, potato variety, and frying method. This approach integrates multiple response variables into a single numerical value called desirability, which varies between 0 (minimum desirability) and 1 (optimal). The most notable treatment corresponded to the Bicentenaria variety, fried using the DF method, with an ultrasound power of 28.93% and a temperature of 77.10°C, achieving the highest desirability (0.884). These conditions allowed a favorable balance to be obtained between the variables evaluated, reflected in a low content of AC (19 µg/kg) and GA (6.971 mg/kg), together with an adequate hardness (7.5 N) and acceptable color quality (Δ*E* = 14.83).

**TABLE 12:** Optimized treatments through desirability analysis.

Variable	Optimal treatments		
	OT1	OT2	OT3
Power (%)	28.93	22.06	20.10
Precooking (°C)	77.10	73.60	76.21
Variety	Bicentenaria	Única	Bicentenaria
Method	Deep‐frying	Oven‐frying	Oven‐frying
Δ*E*	14.83	15.25	14.95
Hardness (*N*)	7.50	7.62	7.64
Acrylamide (µg/kg)	19.00	34.00	17.00
Glycoalkaloids (mg/kg)	6.971	0.059	9.180
Desirability	0.88	0.863	0.849

*Note:* Δ*E*: color variation.

Abbreviations: OT1, optimal treatment 1; OT2, optimal treatment 2; OT3, optimal treatment 3.

Staged frying methods, in which high initial temperatures are followed by lower temperatures, have been shown to reduce AC levels by approximately 50% [49]. However, the AC content in fried potato products tends to be highly variable, with control values ranging from 785 to 964 µg/kg depending on the frying method used [50, 51]. Even reported extremely high levels of 5021 µg/kg in White Rose potatoes fried at 165°C, while lower values were found in other varieties, ranging from 358 to 646 µg/kg.

In contrast, the results of this study show that the combined application of ultrasound and controlled precooking significantly reduced AC levels in the varieties evaluated. In particular, the Bicentenaria variety reached a value of 19 µg/kg, and the Única variety reached 34 µg/kg, using precooking temperatures in the range of 73.6°C–77.1°C, confirming the effectiveness of the process. According to Koyuncu & Duran [11], temperature control is a determining factor in AC mitigation, with 175°C being the optimal condition for the frying process. However, in this study, temperatures of 180°C for 6 min were used in DF and 200°C for 20 min in OF, also obtaining low AC values, which demonstrates the effectiveness of the conditions applied in combination with the pretreatments.

The optimal treatment was experimentally validated in triplicate, obtaining values of 18.30 ± 1.20 µg/kg for AC, 6.89 ± 0.41 mg/kg for α‐chaconine, 7.42 ± 0.35 N for hardness, and 14.91 ± 0.28 for Δ*E*, which showed no significant differences (*p* > 0.05) from the predicted values, confirming the validity of the model.

## 4. Conclusion

The study demonstrates that ultrasound**-**assisted pretreatment, combined with precooking and DF at controlled temperatures, effectively reduces AC while maintaining safe levels of GA. Taguchi and RSM were used to optimize the processing conditions, identifying parameters that maximize product safety and quality. These findings provide practical guidance for safer and more efficient industrial potato processing.

## Author Contributions

Pedro G. Vargas‐Finaflor: conceptualization, methodology, formal analysis, investigation, writing–original draft, visualization, funding acquisition, and project administration.

Elfer O. Obispo‐Gavino: project supervision, methodological support, statistical design, validation, and writing–review and editing.

Sergio E. Contreras‐Liza: project supervision, provision of resources, methodological support, and writing–review and editing.

Delsi M. Huaita Acha: writing–review and editing, critical review of the manuscript, editorial support, and contribution to the discussion of results.

Edwin A. Macavilca: methodological supervision, guidance on analytical procedures, and review of methodological implementation.

Abraham G. Ygnacio Santa Cruz: methodological supervision, support in data analysis planning, and technical advice for methodological implementation.

Michelle Lozada‐Urbano: writing–review and editing, critical review of the manuscript, editorial support, and contribution to the discussion of results.

## Funding

This research was funded by the Consejo Nacional de Ciencia, Tecnología e Innovación Tecnológica (CONCYTEC) under Grant Number PE501090202‐2024.

## Conflicts of Interest

The authors declare no conflicts of interest.

## Data Availability

The data that support the findings of this study are openly available in Figshare at https://doi.org/10.6084/m9.figshare.30063037.
